# Frontline ABVD Remains an Effective Option in Advanced-Stage Classical Hodgkin Lymphoma: Real-World Data from Turkey

**DOI:** 10.3390/jcm15145579

**Published:** 2026-07-16

**Authors:** Derya Demirtas, Elif Suyani, Alper Koc, Bilal Aygun, Zeynep Tugba Karabulut, Nurhilal Buyukkurt, Cagatay Unsal, Didar Yanardag Acik

**Affiliations:** 1 Department of Hematology, Adana City Training and Research Hospital, University of Health Sciences, 01230 Adana, Turkey; elifsuyani@hotmail.com (E.S.); bilalaygun777@gmail.com (B.A.); drzeyneptk@gmail.com (Z.T.K.); nurhilalt@yahoo.com (N.B.); cgtyunsal@gmail.com (C.U.); didaryanardag@gmail.com (D.Y.A.); 2 Department of Hematology, Elazıg Fethi Sekin City Hospital, 23280 Elazıg, Turkey; alperkoc44@hotmail.com

**Keywords:** classical Hodgkin Lymphoma, ABVD, real-world outcomes, advanced-stage disease, progression-free survival, overall survival

## Abstract

**Objectives**: Novel agents such as brentuximab vedotin and anti-PD-1 antibodies, together with PET-adapted intensified strategies, have improved outcomes in advanced-stage classical Hodgkin lymphoma (cHL). However, their applicability remains limited in many middle-income countries because of restricted drug availability, reimbursement barriers, and toxicity concerns. Therefore, real-world data on the effectiveness of ABVD (doxorubicin, bleomycin, vinblastine, and dacarbazine) remains essential. **Methods**: We conducted a retrospective two-center study including 95 patients with newly diagnosed cHL treated with first-line ABVD. Baseline characteristics, treatment response, progression-free survival (PFS), overall survival (OS), and mortality were compared between early-stage (I–II, *n* = 33) and advanced-stage (III–IV, *n* = 62) disease. **Results**: The median age was 38 years (range, 18–73), and 69.5% were male; 65% had advanced-stage. Complete response rates were high in both early-stage (97%) and advanced-stage (95.2%) patients. Median PFS (21 vs. 23 months; *p* = 0.994) and OS (23 vs. 25 months; *p* = 0.848) were similar. Five-year OS was 84.3% for early-stage and 98% for advanced-stage patients (*p* = 0.292), while five-year PFS was 55.3% and 29.6% (*p* = 0.526). Mortality rates were comparable. **Conclusions**: In this real-world cohort, first-line ABVD achieved high response rates and favorable long-term survival in advanced-stage cHL, supporting its continued use as a frontline treatment option, particularly in settings where access to novel therapies may be limited.

## 1. Introduction

Hodgkin lymphoma (HL) arises from the malignant transformation of B-lineage cells within the lymphoid system and is classified into two main subtypes: classical Hodgkin lymphoma (cHL) and nodular lymphocyte-predominant Hodgkin lymphoma (NLPHL) [1]. cHL is one of the most frequent lymphomas and is generally considered a highly curable disease with standard ABVD (doxorubicin, bleomycin, vinblastine, dacarbazine) chemotherapy regimen, with consolidative radiotherapy in some cases [1,2]. Since the introduction of the ABVD regimen several decades ago, it has formed the backbone of first-line therapy worldwide [3]. However, despite its success, relapse or refractory disease, especially in advanced stages, and long-term toxicity remain concerns, prompting the development of newer strategies [2].

In recent years, the therapeutic landscape has shifted significantly with the advent of novel agents and intensified regimens [3]. The incorporation of CD30-targeting antibody-drug conjugate therapy plus AVD, as well as immune checkpoint inhibitors such as anti-programmed death-1 (PD-1) antibodies plus chemotherapy, has demonstrated improved disease control in advanced-stage cHL [3,4]. Despite these advances, the integration of novel agents into routine practice remains difficult in some countries due to limited drug access, reimbursement barriers, and the heightened toxicity risks of intensified regimens [5,6]. Consequently, many patients still rely on standard chemotherapy, with ABVD remaining the most accessible option [7]. Yet evidence supporting its real-world effectiveness, especially in resource-limited settings, is still insufficient [7]. Therefore, we aimed to evaluate the real-world effectiveness of first-line ABVD, particularly in advanced-stage cHL, by comparing it with early-stage cHL, where access to novel therapies remains limited.

## 2. Methods

### 2.1. Study Design

This retrospective observational two-center study was conducted at the University of Health Sciences, Adana City Training and Research Hospital, a tertiary referral center in Adana, Turkey, and Elazığ Fethi Sekin City Hospital. Ethical approval was obtained from the local institutional review board, and the study was conducted in accordance with the principles of the Declaration of Helsinki. A total of 115 adult patients diagnosed with cHL between January 2015 and December 2025 were identified through institutional records. Twenty patients were excluded for one or more of the following reasons: initiation of treatment with a regimen other than ABVD, incomplete diagnostic or staging data, lack of response or follow-up information, discontinuation of therapy before response assessment, or age < 18 years. The final study population consisted of 95 eligible patients who received ABVD as first-line therapy.

### 2.2. Data Collection

Demographic data, baseline clinical features, laboratory findings, stage distribution, presence of B symptoms, bulky disease status, extranodal involvement, and treatment details at the time of diagnosis were recorded from the hospital electronic database. All cases had biopsy-proven lymphoma, with histopathological diagnosis confirmed by an experienced hematopathologist, and staging was performed prior to treatment using the modified Ann Arbor system [8]. Routine staging was performed, which included computerized tomography (CT) scans of the chest, abdomen, and pelvis or positron emission tomography/computerized tomography (PET/CT), and all radiologic assessments were reviewed at baseline and after completion of therapy; interim PET findings were recorded when available. Treatment details, dose modifications, bleomycin omission, and information regarding supportive care or radiotherapy were also collected.

### 2.3. Treatment Protocol

All patients received standard ABVD chemotherapy administered on days 1 and 15 of a 28-day cycle. The number of cycles was determined based on disease stage and interim response. According to institutional practice, interim PET/CT was performed after two cycles, and bleomycin was subsequently omitted in patients with a favorable metabolic response. Bleomycin omission or modification was also permitted in cases of suspected pulmonary toxicity. Consolidative radiotherapy was given selectively, typically for early-stage bulky disease or residual PET-avid lesions. Supportive care followed institutional practice guidelines.

### 2.4. Response Assessment

Treatment response was evaluated according to the Lugano classification [9]. All patients underwent baseline, interim, and end-of-treatment PET/CT. Only two patients were additionally assessed with contrast-enhanced CT at the end of treatment. A Deauville score of 1–3 on PET/CT was defined as complete response (CR), whereas a Deauville score of 4–5 was considered refractory disease. The appearance of new lesions with a Deauville score of 4–5 was considered progressive disease. Response rates were compared between the early-stage (I–II) and advanced-stage (III–IV) groups. Relapse or progression was confirmed radiologically.

### 2.5. Outcomes

The primary endpoints of the study were progression-free survival (PFS) and overall survival (OS). PFS was defined as the time from diagnosis to disease progression, relapse, or death from any cause. OS was defined as the time from diagnosis to death from any cause or last clinical follow-up.

### 2.6. Statistical Analysis

All statistical analyses were performed using IBM SPSS Statistics (version 24.0; IBM Corp., Armonk, NY, USA). Continuous variables were expressed as medians with ranges, and categorical variables as counts with corresponding percentages. Between-group comparisons were conducted using the Mann–Whitney U test for continuous variables and the chi-square or Fisher’s exact test, as appropriate, for categorical variables. Survival analyses were generated using the Kaplan–Meier and Cox regression methods and compared using the log-rank test. A *p*-value < 0.05 was considered statistically significant.

## 3. Results

### 3.1. Patients and Characteristics

A total of 115 patients were initially screened, and 95 met the eligibility criteria for inclusion. Of these, 33 patients (34.7%) had early-stage disease (I–II), while 62 (65.3%) had advanced-stage disease (III–IV). When early-stage disease was classified as favorable and unfavorable, 13 patients were in the early favorable stage and 20 patients were in the early unfavorable stage. The median age at diagnosis was 38 years (range: 18–73), and 66% were male. Baseline demographic and clinical characteristics are summarized in Table 1. B symptoms were more common in advanced-stage disease, whereas rates of bulky disease and extranodal involvement did not differ significantly between the groups (Table 1 and Table 2).

### 3.2. Treatment Delivery and Response Rates

All patients received first-line ABVD as planned, and most completed the intended treatment course. In the early-stage group, 8 patients (24%) received two cycles of ABVD, 14 (42%) received four cycles, and 11 (34%) received six cycles. In the advanced-stage group, the majority of patients completed six cycles of ABVD. Only three patients (4.8%) received two cycles and three (4.8%) received four cycles because of primary refractory disease.

The salvage regimens used for refractory or progressive disease were ICE (ifosfamide, carboplatin etoposid, IGEV (ifosfamide, gemcitabine, vinorelbine), brentuximab vedotin (BV)-bendamustin, BV-GDP (gemcitabin, cisplatin, dexamethasone), DHAP (cytarabine, cisplatin, dexamethasone), Gem-Vin (gemcitabine-vinorelbine), BV-ICE, BV, BV-DHAP. Autologous stem cell transplantation was conducted in 6 patients in the early-stage group and 14 patients in the advanced-stage group (Table 3).

Complete response (CR) rates were uniformly high. CR was achieved in 97% of early-stage patients and 95.2% of those with advanced-stage disease, demonstrating no significant difference between the groups (Table 3).

### 3.3. Survival

**Figure 1:**
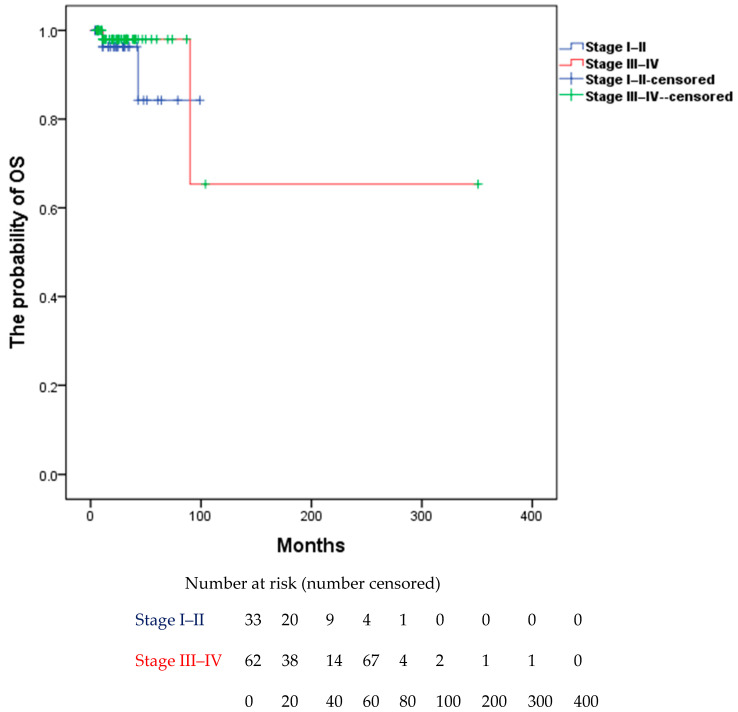
The estimated OS rates in patients with early-stage and advanced-stage HL were 96.3% vs. 98.0% at 2 years, 84.3% vs. 98.0% at 5 years, and 84.3% vs. 65.3% at the end of follow-up (*p* = 0.504); the median OS was not reached in either group.

The median follow-up period was 24 months (range, 4–351 months). The estimated OS rate at the end of follow-up was 84.3% in the early-stage group and 65.3% in the advanced-stage group. The estimated 2-year OS rates were 96.3% vs. 98.0% for the early- and advanced-stage groups, respectively. The estimated 5-year OS rates were 84.3% vs. 98.0%, respectively, with no significant difference between the groups (*p* = 0.504) (Figure 1). The median OS was not reached in either group.

**Figure 2:**
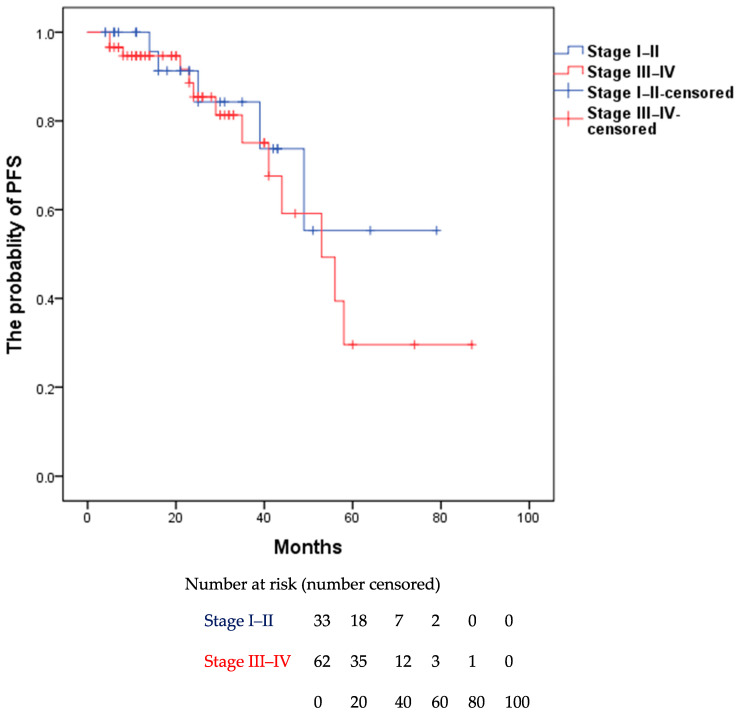
The estimated PFS rates in patients with early-stage and advanced-stage HL were 91.3% vs. 85.4% at 2 years, 55.3% vs. 29.6% at 5 years, and 55.3% vs. 29.6% at the end of follow-up (*p* = 0.526).

The estimated PFS rate at the end of follow-up was 55.3% in the early-stage group and 29.6% in the advanced-stage group. The estimated 2-year PFS rates were 91.3% vs. 85.4% for the early- and advanced-stage groups, respectively. The estimated 5-year PFS rates were 55.3% vs. 29.6%, respectively (*p* = 0.526, 95% CI: 56 (44.8–67.12)) (Figure 2). The median PFS was 56 months in the advanced-stage group, whereas it was not reached in the early-stage group. Stage, sex, presence of B symptoms, lactate dehydrogenase (LDH), albumin, and hemoglobin levels were not significantly associated with either OS or PFS (Table 4 and Table 5).

### 3.4. Mortality Outcomes

Overall mortality within the cohort was low, with early-stage and advanced-stage patients exhibiting similar rates (6.1% vs. 3.2%) (Table 3). No excess mortality attributable to treatment complications was recorded.

## 4. Discussion

The therapeutic landscape of cHL has evolved substantially over the past decade. Landmark trials—most notably ECHELON-1—demonstrated that BV-AVD improved PFS and, with extended follow-up, OS compared to ABVD in stage III–IV disease [10]. Similarly, the emergence of anti-PD-1-based combinations has introduced a significant paradigm shift. In advanced-stage cHL, the phase 3 trial by Herrera et al. [11] showed that nivolumab administered concurrently with AVD resulted in superior PFS and high end-of-treatment metabolic response rates compared with BV-AVD, reinforcing the therapeutic potential of PD-1 blockade in the frontline setting. In addition, the phase 2 study by Lynch et al. [12] suggests that pembrolizumab given concurrently with AVD can induce strong early metabolic responses and appears feasible and well tolerated in patients with cHL, including those with advanced-stage disease. Ultimately, BV and nivolumab have been added to the AVD regimen as an alternative to ABVD in advanced-stage cHL in the final National Comprehensive Cancer Network (NCCN) guideline [13]. Despite the advantages of these novel agents, the high cost of these regimens and the need for infrastructural and supportive-care capacities that are not uniformly available across regions prevent their equitable use worldwide. As highlighted by Barrios et al. [6], this barrier is even more pronounced in low-income and middle-income countries, further underscoring the need for real-world studies that assess patient outcomes when access to novel therapies is limited. Similarly, the high cost of frontline regimens incorporating novel agents, such as BV or nivolumab, remains a major challenge, particularly in countries with limited healthcare resources, including our country. Replacing bleomycin with BV in first-line treatment for advanced-stage HL has been associated with substantially higher treatment costs in several cost-effectiveness analyses [14,15,16]. Nevertheless, Delea et al. [17] reported that BV-AVD was cost-effective as frontline therapy for patients with advanced-stage HL from the perspective of the US healthcare payer. A recent pharmacoeconomic study from China also demonstrated that adding either nivolumab or BV to AVD was a cost-effective strategy, particularly for adolescents and older adults with cHL [18]. Although evidence regarding the cost-effectiveness of nivolumab-AVD remains limited, available economic analyses suggest that nivolumab-AVD may be a more cost-effective option than BV-AVD for patients with advanced-stage classical HL [19]. However, because neither regimen is routinely reimbursed for frontline treatment in our country, this potential economic advantage has had little impact on routine clinical practice.

With respect to toxicity, the ECHELON-1 trial demonstrated that patients treated with BV-AVD experienced significantly higher rates of peripheral neuropathy than those receiving ABVD, although most cases were reversible [10]. Regarding nivolumab, although direct comparative data between nivolumab-AVD and ABVD remain limited, nivolumab-AVD appears to have a more favorable safety profile than BV-AVD [11]. Data regarding pembrolizumab-based frontline therapy remain insufficient to draw definitive conclusions about its comparative toxicity profile.

Another important limitation of the above-mentioned studies is their relatively short follow-up period. Based on this, the survival analysis was performed at nearly two years, which is a short time period for a malignant disease like cHL. And in our clinics, favorable responses to ABVD in advanced-stage cHL attracted our attention. Since we did not have a sufficient number of patients who were treated with novel agents in the frontline setting, we compared the efficacy of ABVD in advanced-stage cHL with early-stage diseases in this retrospective real-world cohort of 95 patients treated uniformly with ABVD. We observed a relatively high CR rate of 95.2% in advanced-stage patients. Also, we observed favorable long-term survival outcomes across both early- and advanced-stage (OS at 5 years; 84% vs. 98%).

Although the level of OS in this cohort was satisfactory, PFS was found to be relatively low in both groups, and even lower in patients with advanced-stage disease, which requires further elucidation. The differences in pharmacokinetics among ethnicities might be an explanation. Although our study did not assess pharmacogenetic determinants or detailed toxicity profiles, potential population-specific differences in treatment tolerance remain an important contextual consideration. Several observational reports from Southern Europe, Asia, and the Middle East suggest that patients in these regions may experience higher rates of hematologic and non-hematologic toxicities with intensified regimens compared with Northern European cohorts [20]. Such variation has been attributed to multiple factors, including differences in comorbidity burden, nutritional status, hepatic enzyme polymorphisms, supportive-care capacity, and other patient-related characteristics [21,22,23]. While these factors were not directly evaluated in our cohort, the variability described in the literature indicates that treatment regimens may not be equally feasible or appropriate across all populations. In this context, real-world analyses—such as ours—are valuable for assessing the practicality and effectiveness of ABVD and also the novel agents within specific regional and demographic settings.

Within this clinical and structural context, our results demonstrate that ABVD remains an effective and well-tolerated regimen even among patients with advanced-stage disease, who constituted two-thirds of our cohort. Complete response rates were over 95% in both stage groups, and 5-year OS remained high, reaching 98% in patients with advanced-stage disease. Although PFS was lower—as expected in real-world advanced-stage HL—the preservation of OS despite lower PFS is consistent with contemporary literature and reflects the availability and effectiveness of salvage therapies, including autologous stem-cell transplantation [24]. Importantly, the absence of a survival disadvantage in advanced-stage patients within our cohort supports the notion that, when access to regimens containing novel agents is limited, ABVD continues to provide durable disease control for the majority of patients [25].

Our study has several strengths, including uniform treatment exposure, a standardized institutional management approach, and comprehensive follow-up data spanning approximately five years. Notably, interim PET imaging was systematically incorporated into clinical decision-making, allowing treatment modification based on metabolic response, largely guided by Deauville criteria. This enhances the real-world relevance of our findings and aligns our approach with contemporary management principles. Nevertheless, certain limitations should be considered. First, the retrospective design and modest sample size may limit the statistical power of subgroup analyses. Second, the absence of detailed toxicity documentation and pharmacogenetic data prevented a more granular assessment of population-specific treatment tolerability.

## 5. Conclusions

Collectively, our results suggest that ABVD remains a feasible frontline treatment option in settings where access to BV-AVD, PD-1-based combinations, or PET-adapted intensified strategies is limited. Future multicenter studies incorporating toxicity profiling, pharmacogenetic data, and economic analyses will be essential to refine treatment strategies further and to ensure that evidence-based yet context-appropriate care is delivered across diverse healthcare environments. These findings reaffirm the ongoing importance of generating region-specific real-world data to inform treatment choices where healthcare resources and drug availability vary widely.

## Figures and Tables

**Table 1:** Baseline demographic and clinical characteristics of patients with cHL.

Characteristics	No of Patients (%)
Total	95
Age (year) (range)	38 (18–73)
Sex (M/F)	66/29
Histology	
Nodular sclerosis	70 (73.7)
Mixed cellularity	12 (12.6)
Lymphocyte rich	11 (11.6)
Lymphocyte depleted	2 (2.1)
Stage	
Stage I	4 (4.2)
Stage II	29 (30.5)
Stage III	21 (22.1)
Stage IV	41 (43.2)
B symptoms	
Yes	65 (68.4)
No	30 (31.6)
Bulky disease	
Yes	3 (3.2)
No	92 (96.8)
IPS (available in 64/95)	
0–2	27 (42)
≥3	37 (58.9)
Number of patients who underwent radiotherapy	8 (8.9)
Spleen involvement	
Yes	22 (23.2)
No	73 (76.8)
Liver involvement	
Yes	8 (8.4)
No	87 (91.6)
Bone marrow involvement	
Yes	24 (25.3)
No	71 (74.7)
Hemoglobin, g/dL, median, (range)	11.6 (6.2–17.9)
Lymphocyte, ×10^3^/µL, median, (range)	1.8 (0.1–7.6)
Albumin, g/dL, median, (range)	3.8 (2.6–4.9)
LDH, u/L	235 (159–1062)

LDH: Lactate Dehydrogenase; IPS: International Prognostic Score.

**Table 2:** Comparison of baseline characteristics between early-stage (I–II) and advanced-stage (III–IV) cHL patients.

Characteristics	Stage I–II (*n* = 33)	Stage III–IV (*n* = 62)	*p*-Value
Age (years) (range)	42 (18–66)	37 (18–73)	0.702
Sex (M/F)	28/5	38/24	0.020
Histology			0.313
Nodular sclerosis	22 (66.6)	48 (77.4)	
Mixed cellularity	5 (15.2)	7 (11.3)	
Lymphocyte rich	6 (18.2)	5 (8.1)	
Lymphocyte depleted	0 (0)	2 (3.2)	
B symptoms			0.000
Yes	8 (24.2)	57 (91.9)	
No	25 (75.8)	5 (8.1)	
Hemoglobin, g/dL, median, (range)	14.3 (9.1–17.8)	11.2 (6.2–17.9)	0.000
Lymphocyte, ×10^3^/µL, median, (range)	1.9 (1.0–3.6)	1.7 (0.1–7.6)	0.113
Albumin, g/dL, median, (range)	4.1 (3.1–4.9)	3.7 (2.6–4.6)	0.000
LDH, u/L	211 (163–449)	245 (159–1062)	0.020

LDH: Lactate Dehydrogenase.

**Table 3:** Treatment response, progression, mortality, and survival outcomes in early-stage versus advanced-stage cHL.

Characteristics	Stage I–II (*n* = 33)	Stage III–IV (*n* = 62)	*p*-Value
Response to treatment, *n* (%)			1.000
CR	32 (97%)	59 (95.2)	
Primary refractory	1 (3%)	3 (4.8%)	
Progression, *n* (%)			0.586
Present	5 (15.6%)	13 (22%)	
Absent	27 (84.4%)	46 (78%)	
The number of deaths	2 (6.1%)	2 (3.2%)	0.608
The number of salvage regimen lines before ASCT, *n* (%)	2	8	1.000
1	4	6	
2			
ASCT, *n*	6	14	1.000

CR: Complete Response; ASCT: Autologous Stem Cell Transplantation.

**Table 4:** Univariate analysis of the effect of stage, sex, presence of B symptoms, LDH, albumin, and hemoglobin levels on OS.

Variable	*p*-Value (Univariate)	HR	95% CI
Stage	0.512	1.938	0.268–14.019
Sex	0.492	0.027	0–777
B symptom	0.749	0.687	0.069–6.857
LDH	0.575	1.002	0.996–1.008
Albumin	0.235	5.378	0.335–86.413
Hemoglobin	0.436	1.198	0.761–1.888

LDH: Lactate Dehydrogenase.

**Table 5:** Univariate analysis of the effect of stage, sex, presence of B symptoms, LDH, albumin, and hemoglobin level on PFS.

Variable	*p*-Value (Univariate)	HR	95% CI
Stage	0.528	0.717	0.255–2.017
Sex	0.325	0.570	0.186–1.745
B symptom	0.267	0.531	0.174–1.625
LDH	0.976	1	0.995–1.005
Albumin	0.240	0.541	0.194–1.507
Hemoglobin	0.382	0.908	0.730–1.128

LDH: Lactate Dehydrogenase.

## Data Availability

The datasets generated and analyzed during the current study were obtained from the hospital records of patients with classic Hodgkin lymphoma at the University of Health Sciences, Adana City Training and Research Hospital. Due to patient confidentiality and institutional regulations, these data are not publicly available but are available from the corresponding author upon reasonable request and with the approval of the institutional ethics committee.
